# Genomic analyses reveal an absence of contemporary introgressive admixture between fin whales and blue whales, despite known hybrids

**DOI:** 10.1371/journal.pone.0222004

**Published:** 2019-09-25

**Authors:** Michael V. Westbury, Bent Petersen, Eline D. Lorenzen

**Affiliations:** 1 Natural History Museum of Denmark, University of Copenhagen, Øster Voldgade, Copenhagen K, Denmark; 2 Centre of Excellence for Omics-Driven Computational Biodiscovery (COMBio), Faculty of Applied Sciences, AIMST University, Kedah, Malaysia; Senckenberg am Meer Deutsches Zentrum fur Marine Biodiversitatsforschung, GERMANY

## Abstract

Fin whales (*Balaenoptera physalus*) and blue whales (*B*. *musculus*) are the two largest species on Earth and are widely distributed across the world’s oceans. Hybrids between these species appear to be relatively widespread and have been reported in both the North Atlantic and North Pacific; they are also relatively common, and have been proposed to occur once in every thousand fin whales. However, despite known hybridization, fin and blue whales are not sibling species. Rather, the closest living relative of fin whales are humpback whales (*Megaptera novaeangliae*). To improve the quality of fin whale data available for analysis, we assembled and annotated a fin whale nuclear genome using in-silico mate pair libraries and previously published short-read data. Using this assembly and genomic data from a humpback, blue, and bowhead whale, we investigated whether signatures of introgression between the fin and blue whale could be found. We find no signatures of contemporary admixture in the fin and blue whale genomes, although our analyses support ancestral gene flow between the species until 2.4–1.3 Ma. We propose the following explanations for our findings; i) fin/blue whale hybridization does not occur in the populations our samples originate from, ii) contemporary hybrids are a recent phenomenon and the genetic consequences have yet to become widespread across populations, or iii) fin/blue whale hybrids are under large negative selection, preventing them from backcrossing and contributing to the parental gene pools.

## Introduction

The fin whale (*Balaenoptera physalus*) is a large species of baleen whale. It can grow up to 26 m long and attain a weight of 60–80 metric tonnes [1]. Like many large rorquals, it is widely distributed across most of the world’s oceans, and is second in size only to the blue whale (*B*. *musculus*) [1]. Despite a divergence time of ~8.35 million years ago (Ma) [2], hybrids between fin and blue whales have been reported since the beginning of early modern whaling, in the late 1800’s [3,4]. However, it was not until the 1990’s that hybrids could be investigated using genetic data. Molecularly confirmed first-generation hybrids have been found in Spain [5], Iceland [4,6,7], and Japanese fish markets [7]. Furthermore, hybrids have been estimated to occur at relatively high frequencies, approximately one in every thousand fin whales, based on a questionnaire given to scientists involved in the inspection or collection of these species at whaling factories [5]. Male hybrids appear to be infertile, due to their small testes [6]. However, the fertility of female hybrids is more uncertain, and a pregnant female hybrid has been reported [4].

**Fig 1:**
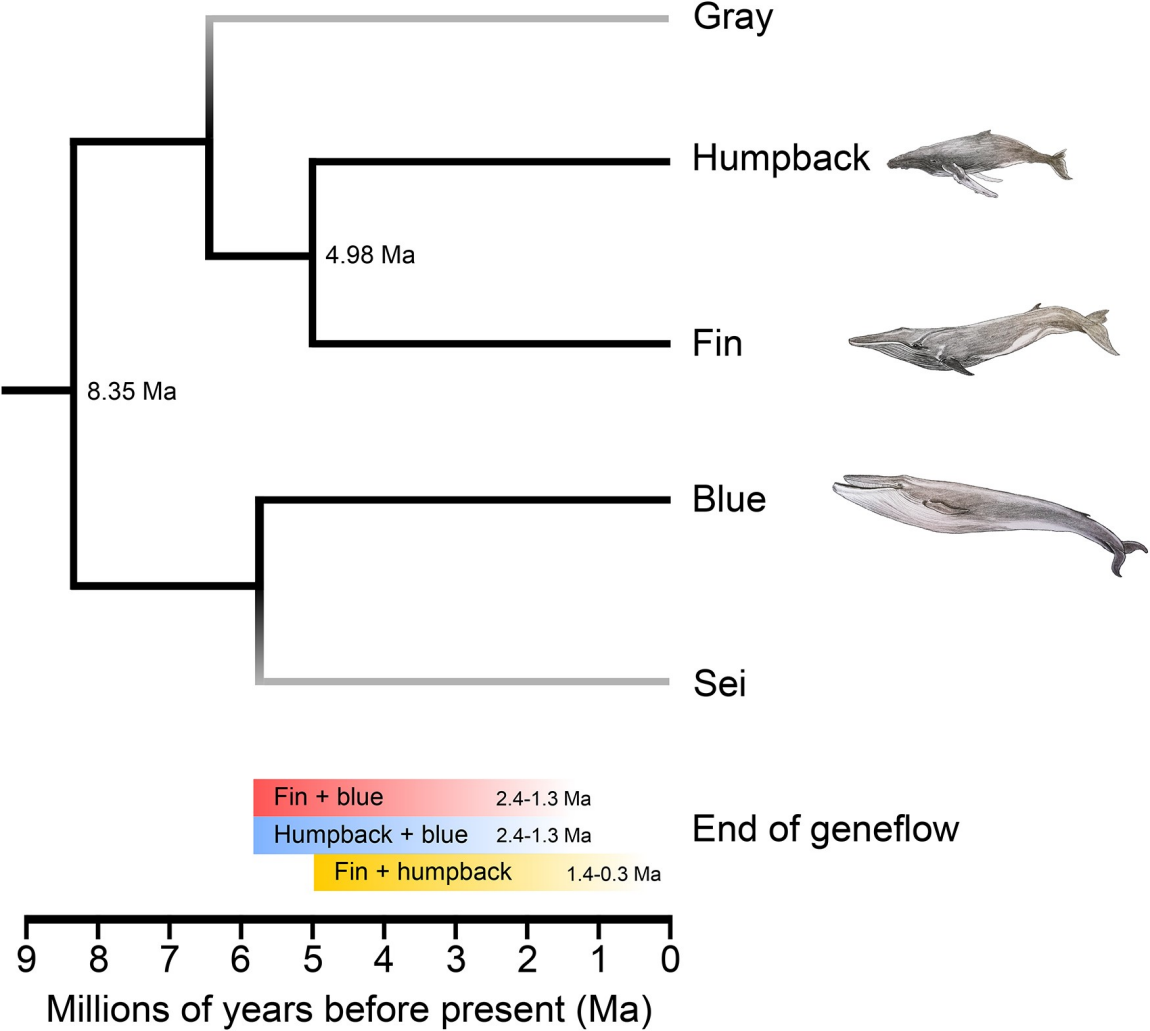
Phylogeny of the three Mysticeti baleen whales analyzed—Fin, humpback, and blue whale, adapted from [2]. Fin+blue whale hybrids are believed to occur at a frequency of one in every 1000 fin whales. In contrast, only one blue+humpback hybrid has been reported. Grey terminal branches indicate species not included in the present study. Illustrations by Binia De Cahsan.

Although putatively fertile hybrids have been recorded between the two species, the fin and blue whale are not sister taxa. Molecular evidence has shown that the closest living relative of the fin whale is the humpback whale (*Megaptera novaeangliae*) [2,8,9] (Fig 1). The discrepancy between genus names reflects that *Megaptera* was coined based on the derived morphological characteristics of the humpback whale [10], and has not been updated with the molecular evidence.

Here, we use publicly available genomic data from four baleen whale species: fin, humpback, blue, and bowhead (*Balaena mysticetus*), to investigate the extent of hybridisation between fin and blue whales, and whether this hybridisation may have occurred long before the first reported hybrid in the 1800’s. To achieve this, we assembled a fin whale nuclear genome, performed three independent admixture analyses, and in the process, assessed the influence of ascertainment bias based on mapping reference selection.

## Methods

### *De novo* assembly and annotations

We assembled the draft nuclear genome of a female fin whale from the U.S. West coast utilising only publically available data. We downloaded ~400bp insert fin whale Illumina reads (SRR935201) [11] from the European Nucleotide Archive (ENA). We trimmed Illumina adapter sequences from reads and removed reads shorter than 30bp using skewer [12]. From these trimmed reads we constructed in-silico mate paired library reads with insert sizes 1kb, 2kb, 5kb, 10kb, and 20kb using the repeatmasked minke whale genome as a reference (GCA_000493695.1) [11] and Cross-Species Scaffolding, specifying default parameters (100bp reads of ~10x coverage) [13]. Specific read numbers and information can be found in S1 Table. We selected the minke whale genome due to its high assembly quality (scaffold N50 ~12.8Mb, contig N50 ~22.7kb) and its relatively close phylogenetic relationship to the fin whale (divergence time of ~10.5 million years), which should retain high levels of synteny between the two species. We prepared the fin whale reads for *de novo* assembly by removing PCR duplicates with prinseq [14], and an error correction step using a kmer size of 31 in tadpole from the bbtools toolsuite [15]. We constructed a *de novo* assembly with these error-corrected reads and the in-silico mate paired libraries using SOAPdenovo2 [16], specifying a kmer size of 41. The short insert reads were used in both the contig construction and scaffolding steps while the mate paired libraries were only used in the scaffolding step. Although the fin whale and minke whale genomes ought to be highly syntenous, especially at the 20kb level, we specified each mate paired library as a different ranking in the SOAP config file to reduce the chances of mis-assemblies brought over by using in-silico mate paired libraries. The shortest insert sizes had higher rankings, meaning that if a longer insert library contradicted the shorter inserts, they were not used for scaffolding. We performed an additional gap closing on the assembly with sealer [17], utilising various kmer sizes (50, 60, 70, 80) and the error-corrected short insert reads. The assembly continuity was assessed using quast v4.5 [18] and gene content was assessed using BUSCOv3 [19] and the mammalian BUSCO gene set database.

### Repeat masking and annotation

Repeats and low complexity DNA sequences were catalogued and masked in the resultant fin whale genome using RepeatMasker version open-4.0.7 [20] using the species repeat database ‘fin’ with RepBase database version 20170127. Remaining specific repetitive elements were predicted *de novo* using RepeatModeler version 1.0.11 [21] from the masked genome. A second round of RepeatMasker was subsequently run with the model generated from RepeatModeler as custom library input on the previously masked genome.

Genome annotation was performed on the repeatmasked genome using the genome annotation pipeline MAKER2 version 2.31.9 [22] with ab-initio and homology-based gene predictions. Protein sequences from killer whale (*Orcinus orca*), beluga whale (*Delphinapterus leucas*), cattle (*Bos taurus)*, dog, (*Canis lupus familiaris*), humans (*Homo sapiens*), minke whale (*Balaenoptera acutorostrata*), and the finless porpoise (*Neophocaena asiaeorientalis*) were used for homology-based gene prediction. As no training gene models were available for the fin whale, we used CEGMA [23,24] to train the ab-initio gene predictor SNAP [25], rather than using the de-novo gene predictor in Augustus [26]. MAKER2 was run with “model_org = simple, softmask = 1, augustus_species = human” and the “snaphmm” parameter was set to the HMM generated in the manual training of SNAP.

### Mapping of other baleen whale genomes

For use in subsequent analyses, we downloaded raw reads for the humpback whale (SRR5665639) and blue whale (SRR5665644) [2], both previously used to investigate ancestral gene flow between rorqual species, and the bowhead whale (SRR1685383) [27] from the ENA. We trimmed Illumina adapter sequences and removed reads shorter than 30bp using skewer [12] and mapped these reads to the fin whale assembly using BWA v0.7.15 [28] and the mem algorithm. We then parsed the output and removed PCR duplicates and reads with a mapping quality less than 30 with SAMtools v1.6 [29]. Furthermore, to investigate for the presence of ascertainment bias caused by mapping to an ingroup species (i.e. the fin whale), we repeated the above steps using the bowhead whale genome (http://www.bowhead-whale.org) as the mapping reference.

### Admixture analyses

We performed three independent analyses to investigate for signatures of admixture between the fin and blue whale based on the known species tree (Fig 1): D-statistics, pairwise distances, and F1 hybrid pairwise sequentially Markovian coalescent model (hPSMC). To differentiate signs of admixture from incomplete lineage sorting between the fin and blue whale, we included the humpback whale as a comparative control. All analyses were repeated twice, once with all individuals mapped to the fin whale genome and once with all individuals mapped to the bowhead whale genome.

### D-statistics

We investigated for signs of unequal shared derived alleles between the blue whale and the fin or humpback whale by performing D-statistics with ANGSD v0.921 [30]. D-statistics works based on a predefined species tree which uses three ingroup and one outgroup taxa. This topology can be written as [[[H1,H2],H3],O] where H1 and H2 are more closely related to one another than either are to H3. The method scans across the genome to find regions that contradict the known species tree, either due to incomplete lineage sorting or admixture between H3 and H1 or H2. An equal occurrence of the topologies [[[H1,H3],H2],O] and [[[H3,H2],H1],O] is most commonly interpreted as incomplete lineage sorting, but can also be caused by equal amounts of gene flow between H3+H1 and H3+H2. Any deviation from this ratio is considered as differential gene flow between the ingroup species analysed. As the known species tree is [[[fin, humpback], blue], outgroup], we are presented with the perfect opportunity to test for admixture between the fin whale and blue whale using D-statistics.

We called bases using a consensus base call (-doAbbababa 2), only considered scaffolds over 100kb in length, specified the bowhead whale as the outgroup, and applied the following filters; minimum base quality of 25 (-minQ 25), minimum mapping quality of 25 (-minMapQ 25), only consider reads that map uniquely to one location (-uniqueOnly 1), remove reads deemed “bad” by ANGSD (-remove_bads 1), and specify window size as 1MB (-blocksize). This was repeated twice, once with all three species mapped to the fin whale genome, and once with all three species mapped to the bowhead whale genome.

ANGSD performs all possible combinations, but we only considered the output with fin and humpback whales as H1 and H2, respectively, and the blue whale as H3, as this is the known species tree [2]. Any other combination would go against the species tree, producing false signs of admixture driven by more recent common ancestry, as opposed to true admixture. To investigate the significance of our results, we performed a weighted block jackknife test using 5 Mb non-overlapping blocks. D values that differed more than three standard errors from zero (|Z| <3) were considered as statistically significant.

### Sliding windows pairwise distances

We conducted three independent 100kb non-overlapping sliding window pairwise distance comparisons (fin vs. humpback, fin vs. blue, humpback vs. blue). This was repeated twice, once with all three species mapped to the fin whale genome, and once with all three species mapped to the bowhead whale genome. We calculated average pairwise distances from the windows using a consensus base call in ANGSD (-doIBS 2), only considering sites found in all three species (-minInd 3), windows that contained at least 75kb data, and the filters; minimum base quality of 25 (-minQ 25), minimum mapping quality of 25 (-minMapQ 25), only consider reads that map uniquely to one location (-uniqueOnly 1), remove reads deemed “bad” by ANGSD (-remove_bads 1). We constructed the non-overlapping 100kb sliding windows using bedtools [31], only considering scaffolds over 100kb in length. After filtering, 12,345 windows of at least 75kb of data in all three species remained when individuals were mapped to the fin whale, and 17,533 windows remained when individuals were mapped to the bowhead whale.

### hPSMC

To investigate whether gene flow ceased at different time periods between the fin and blue whale, as opposed to the humpback and blue whale, we used hPSMC [32]. This was repeated twice, once with all three individuals mapped to the fin whale and once with all three individuals mapped to the bowhead whale. We constructed haploid consensus sequences for the three independent species using ANGSD by considering the base with the highest effective base depth and the following quality filters; minimum base quality of 25 (-minQ 25), minimum mapping quality of 25 (-minMapQ 25), only consider reads that map uniquely to one location (-uniqueOnly 1), remove reads deemed “bad” by ANGSD (-remove_bads 1), only consider sites with at least 5x coverage (-setMinDepthInd 5). We then merged these haploid consensus sequences using the hPSMC toolsuite [32] into a pseudo-diploid sequence and ran it through PSMC [33]. We assumed the generation times of each whale as follows; blue whale—30.8 years, humpback whale—21.5 years, and fin whale—25.9 years [2].

To calibrate the hPSMC plots, we estimated the mutation rates of the fin, humpback and blue whales using the genomes mapped to the fin whale. To calculate the mutation rates of the fin and humpback whale, we performed a genome-wide pairwise distance analysis between the fin and humpback whale using ANGSD. From this, we calculated the mean number of substitutions per year assuming a divergence time between fin and humpback whales of 4.98 Ma [2]. We also estimated the mutation rate in the blue whale by performing genome-wide comparisons between the fin, humpback and blue whale and assuming a divergence time of 8.35 Ma [2]. We calculated the average genome-wide pairwise distance between the fin and humpback whale to be 0.0105. Using a divergence time between the two species of 4.98Ma [2], we estimated a mutation rate of ~1.05x10^-9^ per year. We calculated the average genome-wide pairwise distance between the fin and blue whale and the humpback and blue whale to be the same (0.0128). Using a divergence time of 8.35 Ma [2], we estimated a mutation rate of ~7.7x10^-10^ per year.

The average of the generation times and the mutation rates calculated above were used to calibrate the hPSMC. When comparing the fin/humpback/blue whale we used a generation time of 26.1 years, a mutation rate of 7.7x10^-10^ per year, and therefore a mutation rate of 2x10^-8^ per generation. When comparing the fin/humpback whale, we used a generation 23.7 years, a mutation rate of 1.05x10^-9^ per year, and therefore a mutation rate of 2.49x10^-8^ per generation. From the fin+humpback whale hPSMC output, we manually estimated the pre-divergence Ne (the Ne prior to the exponential increase in diversity) to be ~64,000 individuals. We did this by outputting the text file (-R) using the plot perl script from the PSMC toolsuite and looking into the output text file. We then ran simulations using this pre-divergence Ne while specifying various divergence times between 0 and 3 Ma in 100,000 year intervals using ms [34]. From the fin+blue and humpback+blue whale outputs we estimated the pre-divergence Ne to be ~90,000 individuals in both cases. We then ran simulations using this pre-divergence Ne while specifying various divergence times between 1 and 5 Ma in 100,000 year intervals using ms. Results were plotted and the simulations with an exponential increase in Ne closest to but not overlapping the real data, within 1.5x and 10x of the pre-divergence Ne, were taken as the time interval in which gene flow stopped between the two specified species.

## Results

### Fin whale assembly and annotation

We assembled 2,460,448,386bp (2,025,416,608bp excluding missing data -Ns) of a female fin whale’s nuclear genome in 59,639 scaffolds with a scaffold N50 of 871.4kb, utilising only publically available data and Cross-Species Scaffolding using the minke whale (S2 Table). Initial investigations into the gene content of the assembled genome using BUSCOv3 and the mammalian BUSCO dataset revealed 88.4% complete BUSCOs (S3 Table). Repeat profiling found the genome to consist of 31.49% repetitive elements (S4 Table). We identified a total of 20,335 protein coding genes through genome annotations with MAKER2 [22].

### Mapping of other baleen whale genomes

The mapping results of all individuals included in this study to both the fin whale and bowhead whale genomes can be found in S5 Table.

### Admixture between fin and blue whales

We investigated for signs of admixture between the fin and blue whale based on the known species tree by conducting three independent analyses. Each analysis was repeated twice, once with all individuals mapped to the fin whale genome and once with all individuals mapped to the bowhead whale genome.

First, we investigated for signs of unequal shared derived alleles between the blue whale and either the fin or humpback whale by performing D-statistics. An excess of shared derived alleles between two species could be indicative of admixture between these species. When using the fin whale genome as the mapping reference, we found slightly more derived alleles shared between the fin and blue whale (1,374,331) compared to the humpback and blue whale (1,366,906). This result gave a D score of -0.0027. However, as the Z score was between -3 and 3 (-1.94), this result was deemed non-significant. In contrast, when using the bowhead whale as the mapping reference, we found more derived alleles shared between the humpback and blue whale (1,586,937) compared to the fin and blue whale (1,559,793), giving a D score of 0.0086 and a significant Z score (6.32).

Second, we conducted three independent 100kb non-overlapping sliding window pairwise distance comparisons (fin vs. humpback, fin vs. blue, humpback vs. blue) and plotted the results, looking for any differences between the supposedly equidistant fin vs. blue, and humpback vs. blue, comparisons. A relative excess of highly similar windows could indicate admixture between the species in question. After filtering, 12,345 windows of at least 75kb of data in all three species remained when mapping to the fin whale, and 17,533 when mapping to the bowhead whale.

**Fig 2:**
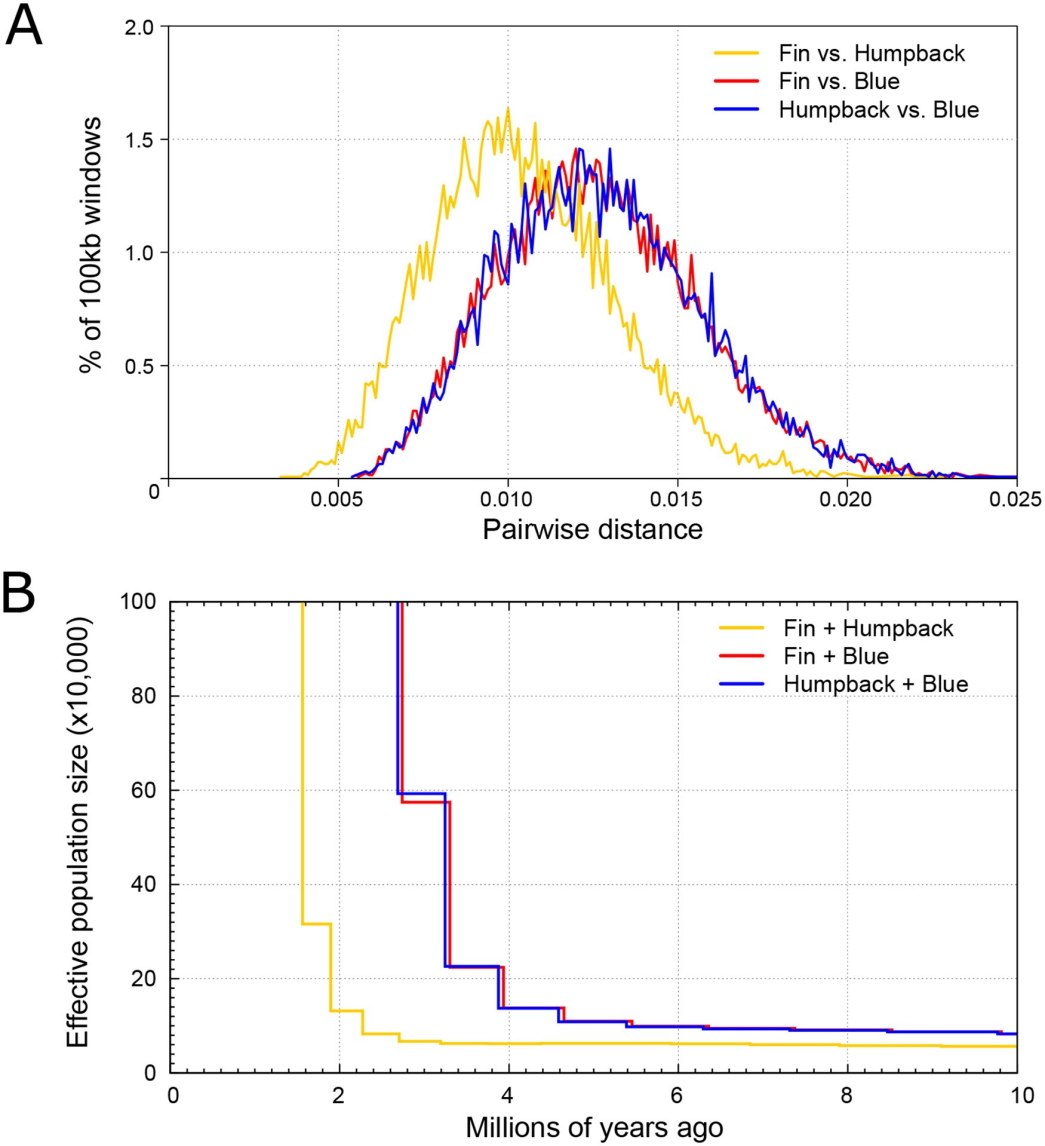
Admixture comparisons performed using the fin whale as the mapping reference. A: Sliding window pairwise comparisons. 100kb non-overlapping window identity-by-state, pairwise distance comparisons between each species pair. B: hPSMC plot based on the demographic history of pseudodiploid sequences constructed from different species pairs.

Regardless of whether the fin whale or the bowhead whale was used as the mapping reference, plots of the 100kb sliding window pairwise distances between the fin vs. blue whale, almost perfectly overlap with that of the humpback vs. blue whale (Fig 2A and S1 Fig). Furthermore, the genome-wide pairwise distance between the fin vs. humpback whale was, on average, less than both the fin vs. blue whale and the humpback vs. blue whale comparisons. This result is concordant with the phylogenetic relationship of the three species (Fig 1).

Our third analysis for signs of admixture between the fin and blue whale was hPSMC. This analysis makes use of the shared demographic history of the species to uncover the approximate point at which gene flow ceased between the two target individuals. We find that the demographic history of the fin/blue whale pseudodiploid genome is near identical to that produced by the humpback/blue whale (Fig 2B and S2 Fig), regardless of which mapping reference genome was used, indicating that gene flow ceased between the blue whale and the other two species at the same time. This is the result one would expect if there was no recent admixture among any of the species. Furthermore, gene flow between the fin and humpback whales ceased more recently than between both the fin and blue whale, and the humpback and blue whale. Similar to the sliding window analysis, this result is concordant with the phylogenetic relationship of the three species (Fig 1). PSMC is known to portray rapid changes in ancestral effective population size as more gradual transitions, and one therefore cannot apply a purely qualitative approach to estimating the divergence time between populations by using the increase in inferred ancestral population size only. Therefore, to gain a better understanding of when gene flow ceased, we ran simulations specifying various divergence times between the species of interest. Our simulations suggest that gene flow ceased between fin and blue whale (S3 Fig), and humpback and blue whale (S4 Fig) 2.4–1.3 Ma (Fig 1). Gene flow between fin and humpback whales ceased later, 1.4–0.3 Ma (Fig 1 and S5 Fig).

## Discussion

A recent study investigating the nuclear genomes of a number of rorqual whale species suggested that the evolutionary history of rorquals occurred in the presence of introgressive gene flow among species [2]. This gene flow most likely occurred when early rorqual lineages began to diverge from one another. The limited genetic differentiation between these hybridising early species could have inhibited outbreeding depression, allowing offspring to survive and pass on their DNA to future generations. Here, we investigated for the presence of introgressive gene flow between two highly diverged (~8.35 Ma) rorqual species, the fin and blue whale, which are currently known to hybridize in the wild [4–7].

Our D-statistics analyses show conflicting results depending on which mapping reference genome was used. We find no signal of introgressive gene flow in the fin and blue whale genomes when using the humpback whale to control for incomplete lineage sorting and the fin whale as the mapping reference. However, when using the bowhead whale as the mapping reference, we find a significant D-statistic result indicating higher levels of admixture between the humpback and blue whale than the fin and blue whale. These contrasting results could be due to mapping reference ascertainment bias, as fin whale-like reads are more likely to map to the fin whale genome than to the bowhead whale genome, artificially shifting the signals of admixture towards the fin whale. Regardless of ascertainment bias, both results are unexpected; viable fin and blue whale hybrids have been reported since the beginning of whaling in the 1800’s [3] and are thought to occur relatively frequently [5], suggesting the two species can and do readily hybridize contemporarily. However, as D-statistics is a relative test, our results could also be explained by humpback/blue whale hybrids occurring at a similar or higher frequency than fin/blue whale hybrids. Although not molecularly confirmed, one putative blue/humpback whale hybrid calf from French polynesia was reported in the late 1990s based on photographic evidence. The individual was diagnosed based on the following characteristics; it was larger than other calves of approximately the same age, it displayed unusual pigmentation (a more uniform speckled gray/blue pattern, reminiscent of blue whales), the pectoral flippers were shorter and more pointed than a humpback’s (with fewer protuberances on the leading edge), the trailing edge of the tail seemed straighter and less ‘scalloped’ than other humpbacks (more similar to *Baleanoptera*), the head was more sharply pointed than is usually seen on humpback whales, the dorsal fin was substantially taller and much more erect than other humpbacks, resembling the dorsal fin of a sei whale, and the number of throat pleats (> 44) was nearly two times greater than those of other humpback whales observed and photographed in the area (< 24) [35].

In contrast to our D-statistics results, our sliding window pairwise distance analysis shows no indication of recent admixture between any of the species, regardless of the mapping reference used. However, these seemingly conflicting results could be explained by ancestral gene flow. Even if gene flow occurred in the past, recombination may have broken up stretches of conspecific DNA, resulting in stretches much shorter than 100kb. D-statistics is much more sensitive to this sort of ancestral admixture, which may have been retained in the humpback whale gene pool at higher frequency due to random chance (i.e genetic drift).

To provide more context into these contemporary fin/blue whale hybrids, we investigated signs of ancestral gene flow between all three species pairs, by performing pseudodiploid fin+blue, humpback+blue, and fin+humpback whale joint demographic analyses using hPSMC. We find evidence for the occurrence of ancestral gene flow between the fin+blue whale, and the humpback+blue whale. Using simulation analyses, we find that gene flow ceased between these species pairs 2.4–1.3 Ma (Fig 2B and S3 and S4 Figs). This was another unexpected result, as this is after fin and humpback whales diverged (~4.98 Ma), and implies that similar amounts of gene flow continued between both humpback+blue whales, and fin+blue whales, for 3.8–2.5 million years after divergence. This also implies that gene flow between fin+blue whales and humpback+blue whales ceased at the same time, which seems improbable. An alternative explanation is that gene flow continued to occur at a relatively high rate between fin and humpback whales after divergence, leading to the indirect transmission of genetic material from blue whales, through fin whales, to humpback whales, as opposed to a direct transfer. This theory is supported by our pseudodiploid fin+humpback whale hPSMC analysis; we find gene flow to have ceased between the fin and humpback whale more recently, 1.4–0.3 Ma (Fig 1 and S5 Fig).

In contradiction with our finding that gene flow ceased between fin and blue whales >1.3 Ma, Árnason et al 2018 briefly mentioned that current hybridization between fin and blue whales has left genome-wide signals of introgression [2]. This interpretation appears to have been based on analyses among fin, blue and gray whales. However, Árnason et al 2018 also find a high proportion of gene flow between the ancestral fin/humpback whale and the blue whale. This may falsely have led to the signal of gene flow reported between fin and blue whales. A more parsimonious explanation is that gene flow occurred after the divergence of gray whales from the fin/humpback ancestor 7.49 Ma [2], but before the divergence of fin and humpback whales 4.98 Ma. Our hPSMC analysis investigating the joint demographic histories of the three species pairs unravels this, and provides an estimate of the approximate time when gene flow may have ended between the species of interest. Our results add another layer and show that the genome-wide signals of introgression in fin whales are most likely remnants of ancestral gene flow, as opposed to contemporary gene flow.

Through the use of a newly assembled fin whale genome and the previously published bowhead whale genome [27], we were able to assess ascertainment bias caused by the mapping reference. While the overall conclusion did not change, our D-statistics results differed slightly, uncovering higher levels of gene flow between the humpback and blue whale than the fin and blue whale. Without the additional two analyses for recent admixture (sliding window pairwise distances and hPSMC), these results could have been interpreted in vastly different ways, simply due to the reference genome used. This result shows the importance of selecting the appropriate reference genome, even with high-coverage data, for admixture analyses. On top of its use here, the new fin whale assembly can have a wide range of applications for future studies, including acting as a mapping reference for future population genomic studies, or supplying gene information for use in comparative genomics. However, as the assembly was scaffolded using *in silico* mate-pair libraries generated by a closely related species, it also has some limitations. The assembly could contain some mis-assemblies caused by changes in the genomic architecture of the fin and minke whale after they diverged ~10.5Ma [2] meaning it may be inadequate for the study of gene copy number variation, chromosomal structural variation, and synteny between species [13].

## Conclusion

Our finding of no continual introgressive gene flow between fin and blue whales could have several explanations, linked to both the data used and the biology of the species investigated. First, fin/blue whale hybrids may not occur between the populations our samples originated from. The fin whale individual used in this study is from the North Pacific, where putative hybrids have been reported. The origin of the blue whale is unknown and it could be from a region where hybridization does not occur. To further investigate whether this is the cause of our finding, more individuals of known origin would need to be investigated. Second, hybrids may only be a recent phenomenon, spurred by commercial whaling. Both fin and blue whales suffered large declines in their population sizes during commercial whaling [36], and may therefore have struggled to find conspecific mates. This recent time frame, coupled with the long life expectancies and generation times of both species [1,37], could prevent the signal of recent hybridisation in the gene pools of the parental species. Finally, the fin/blue whale hybrids may be under such strong negative selection that they do not successfully contribute to the gene pool of future generations. If this scenario is the case, then the reproductive fitness of both species may be suffering from these hybridisation events, and may be of concern for the long-term survival of either species.

## Supporting information

S1 Table Short read libraries used to assemble the fin whale nuclear genome. (DOCX)

S2 Table Fin whale *de novo* assembly quality information recovered using QUAST results on the pre and post scaffolded assembly. (DOCX)

S3 Table BUSCO scores of the fin whale genome assembly when using the BUSCOv3 mammal dataset. (DOCX)

S4 Table Fin whale genome repeat profile. (DOCX)

S5 Table Mapping statistics of all individuals included in the present study mapped to both our fin whale assembly and the previously published bowhead whale genome. Coverage was calculated using the total number of bp in each assembly excluding missing data (Fin whale—2,025,416,608bp, Bowhead whale—2,099,136,199bp).
(DOCX)

S1 Fig Sliding window pairwise comparisons produced using the bowhead whale as the mapping reference. 100kb non-overlapping window identity-by-state, pairwise distance comparisons between each species pair.
(DOCX)

S2 Fig hPSMC plot based on the demographic history of pseudodiploid sequences constructed from different species pairs using the bowhead whale as the mapping reference. (DOCX)

S3 Fig hPSMC plot between the fin and blue whale and simulations of various different divergence times. Greyed out regions represent 1.5x and 10x the pre-divergence effective population size, grey lines represent the simulated data in 100kya intervals starting from 1Ma and ending at 5Ma, black line represents the simulations closest to the real data without overlapping it, red line represents the hPSMC result.
(DOCX)

S4 Fig hPSMC plot between the humpback and blue whale and simulations of various different divergence times. Greyed out regions represent 1.5x and 10x the pre-divergence effective population size, grey lines represent the simulated data in 100kya intervals starting from 1Ma and ending at 5Ma, black line represents the simulations closest to the real data without overlapping it, green line represents the hPSMC result.
(DOCX)

S5 Fig hPSMC plot between the fin and humpback whale and simulations of various different divergence times. Greyed out regions represent 1.5x and 10x the pre-divergence effective population size, grey lines represent the simulated data in 100kya intervals starting from 0Ma and ending at 3Ma, black line represents the simulations closest to the real data without overlapping it, blue line represents the hPSMC result.
(DOCX)

## References

[1] Aguilar A, García-Vernet R. Fin Whale: *Balaenoptera physalus* In: Würsig B, Thewissen JGM, Kovacs KM, editors. Encyclopedia of Marine Mammals (Third Edition). Academic Press; 2018 p. 368–71.

[2] Árnason Ú, Lammers F, Kumar V, Nilsson MA, Janke A. Whole-genome sequencing of the blue whale and other rorquals finds signatures for introgressive gene flow. Sci Adv. 2018 4;4(4):eaap9873. 10.1126/sciadv.aap9873 PMC5884691 29632892

[3] Cocks AH. The fin whale fishery of 1886 on the Lapland coast. Zoologist. 1887;11:207–22.

[4] Spilliaert R, Vikingsson G, Arnason U, Palsdottir A, Sigurjonsson J, Arnason A. Species hybridization between a female blue whale (*Balaenoptera musculus*) and a male fin whale (B. physalus): molecular and morphological documentation. J Hered. 1991;82(4):269–74. 10.1093/oxfordjournals.jhered.a111085 1679066

[5] Bérubé M, Aguilar A. A new hybrid between a blue whale, *Balaenoptera musculus*, and a fin whale, *B*. *physalus*: frequency and implications of hybridization. Mar Mamm Sci. 1998;14(1):82–98.

[6] Arnason U, Spilliaert R, Pálsdóttir A, Arnason A. Molecular identification of hybrids between the two largest whale species, the blue whale (*Balaenoptera musculus*) and the fin whale (*B*. *physalus*). Hereditas. 1991;115(2):183–9. 10.1111/j.1601-5223.1991.tb03554.x 1687408

[7] Cipriano F, Palumbi SR. Genetic tracking of a protected whale. Nature. 1999 1 28;397:307.

[8] Hatch LT, Dopman EB, Harrison RG. Phylogenetic relationships among the baleen whales based on maternally and paternally inherited characters. Mol Phylogenet Evol. 2006;41(1):12–27. 10.1016/j.ympev.2006.05.023 16843014

[9] Steeman ME, Hebsgaard MB, Fordyce RE, Ho SYW, Rabosky DL, Nielsen R, et al Radiation of extant cetaceans driven by restructuring of the oceans. Syst Biol. 2009;58(6):573–85. 10.1093/sysbio/syp060 20525610 PMC2777972

[10] Nowak RM, Walker EP. Walker’s Mammals of the World. Vol. 1 JHU Press; 1999.

[11] Yim H-S, Cho YS, Guang X, Kang SG, Jeong J-Y, Cha S-S, et al Minke whale genome and aquatic adaptation in cetaceans. Nat Genet. 2014;46(1):88–92. 10.1038/ng.2835 24270359 PMC4079537

[12] Jiang H, Lei R, Ding S-W, Zhu S. Skewer: a fast and accurate adapter trimmer for next-generation sequencing paired-end reads. BMC Bioinformatics. 2014;15:182 10.1186/1471-2105-15-182 24925680 PMC4074385

[13] Grau JH, Hackl T, Koepfli K-P, Hofreiter M. Improving draft genome contiguity with reference-derived *in silico* mate-pair libraries. Gigascience. 2018;7(5):giy029. 10.1093/gigascience/giy029 PMC5967465 29688527

[14] Schmieder R, Edwards R. Quality control and preprocessing of metagenomic datasets. Bioinformatics. 2011;27(6):863–4. 10.1093/bioinformatics/btr026 21278185 PMC3051327

[15] Bushnell B. BBTools software package. URL http://sourceforge%20net/projects/bbmap. 2014;

[16] Luo R, Liu B, Xie Y, Li Z, Huang W, Yuan J, et al SOAPdenovo2: an empirically improved memory-efficient short-read de novo assembler. Gigascience. 2012;1(1):18 10.1186/2047-217X-1-18 23587118 PMC3626529

[17] Paulino D, Warren RL, Vandervalk BP, Raymond A, Jackman SD, Birol I. Sealer: a scalable gap-closing application for finishing draft genomes. BMC Bioinformatics. 2015;16:230 10.1186/s12859-015-0663-4 26209068 PMC4515008

[18] Gurevich A, Saveliev V, Vyahhi N, Tesler G. QUAST: quality assessment tool for genome assemblies. Bioinformatics. 2013;29(8):1072–5. 10.1093/bioinformatics/btt086 23422339 PMC3624806

[19] Waterhouse RM, Seppey M, Simão FA, Manni M, Ioannidis P, Klioutchnikov G, et al BUSCO applications from quality assessments to gene prediction and phylogenomics. Mol Biol Evol. 2017;35(3):543–8. 10.1093/molbev/msx319 PMC5850278 29220515

[20] Smit AFA, Hubley R, Green P. RepeatMasker Open-4.0. 2013–2015.

[21] Smit AFA, Hubley R. RepeatModeler Open-1.0. 2008–2015. http://www.repeatmasker.org

[22] Holt C, Yandell M. MAKER2: an annotation pipeline and genome-database management tool for second-generation genome projects. BMC Bioinformatics. 2011;12:491 10.1186/1471-2105-12-491 22192575 PMC3280279

[23] Parra G, Bradnam K, Korf I. CEGMA: a pipeline to accurately annotate core genes in eukaryotic genomes. Bioinformatics. 2007;23(9):1061–7. 10.1093/bioinformatics/btm071 17332020

[24] Parra G, Bradnam K, Ning Z, Keane T, Korf I. Assessing the gene space in draft genomes. Nucleic Acids Res. 2009;37(1):289–97. 10.1093/nar/gkn916 19042974 PMC2615622

[25] Korf I. Gene finding in novel genomes. BMC Bioinformatics. 2004;5:59 10.1186/1471-2105-5-59 15144565 PMC421630

[26] Stanke M, Waack S. Gene prediction with a hidden Markov model and a new intron submodel. Bioinformatics. 2003;19:215–25. 10.1093/bioinformatics/btg1080 14534192

[27] Keane M, Semeiks J, Webb AE, Li YI, Quesada V, Craig T, et al Insights into the evolution of longevity from the bowhead whale genome. Cell Rep. 2015;10(1):112–22. 10.1016/j.celrep.2014.12.008 25565328 PMC4536333

[28] Li H, Durbin R. Fast and accurate short read alignment with Burrows–Wheeler transform. Bioinformatics. 2009;25(14):1754–60. 10.1093/bioinformatics/btp324 19451168 PMC2705234

[29] Li H, Handsaker B, Wysoker A, Fennell T, Ruan J, Homer N, et al The Sequence Alignment/Map format and SAMtools. Bioinformatics. 2009;25(16):2078–9. 10.1093/bioinformatics/btp352 19505943 PMC2723002

[30] Korneliussen TS, Albrechtsen A, Nielsen R. ANGSD: Analysis of Next Generation Sequencing Data. BMC Bioinformatics. 2014;15:356 10.1186/s12859-014-0356-4 25420514 PMC4248462

[31] Quinlan AR. BEDTools: The Swiss-Army Tool for Genome Feature Analysis. Curr Protoc Bioinformatics. 2014;47:11.12.1–34. 10.1002/0471250953.bi1112s47 PMC4213956 25199790

[32] Cahill JA, Soares AER, Green RE, Shapiro B. Inferring species divergence times using pairwise sequential Markovian coalescent modelling and low-coverage genomic data. Philos Trans R Soc Lond B Biol Sci. 2016;371(1699):20150138 10.1098/rstb.2015.0138 27325835 PMC4920339

[33] Li H, Durbin R. Inference of human population history from individual whole-genome sequences. Nature. 2011;475(7357):493–6. 10.1038/nature10231 21753753 PMC3154645

[34] Hudson RR. Generating samples under a Wright–Fisher neutral model of genetic variation. Bioinformatics. 2002;18(2):337–8. 10.1093/bioinformatics/18.2.337 11847089

[35] Poole MM, Darling J. Occurrences of humpback whales in French Polynesia. In: Proc Bienn 13th Biol Mar Mamm. 1999. p. 150.

[36] Roman J, Palumbi SR. Whales before whaling in the North Atlantic. Science. 2003;301(5632):508–10. 10.1126/science.1084524 12881568

[37] Sears R, Perrin WF. Blue Whale: *Balaenoptera musculus* In: Perrin WF, Würsig B, Thewissen JGM, editors. Encyclopedia of Marine Mammals (Second Edition). London: Academic Press; 2009 p. 120–4.

