# Supplementary material for: Genomic analyses reveal an absence of contemporary introgressive admixture between fin whales and blue whales, despite known hybrids
Source: PLoS One. 2019 Sep 25;14(9):e0222004. doi: 10.1371/journal.pone.0222004 (PMC6760757; doi:10.1371/journal.pone.0222004)
Supplement: S1 Table — (DOCX) [file pone.0222004.s001.docx]

**S1 Table:** Short read libraries used to assemble the fin whale nuclear genome.

| **Library type** | **Insert** | **Number of read pairs** |
| --- | --- | --- |
| Short insert paired end | ~400bp | 578,742,088 |
| In-silico mate-pair | 1kb | 13,756,580 |
| In-silico mate-pair | 2kb | 13,175,593 |
| In-silico mate-pair | 5kb | 12,413,319 |
| In-silico mate-pair | 10kb | 12,063,091 |
| In-silico mate-pair | 20kb | 11,957,048 |
