# Supplementary material for: Genomic analyses reveal an absence of contemporary introgressive admixture between fin whales and blue whales, despite known hybrids
Source: PLoS One. 2019 Sep 25;14(9):e0222004. doi: 10.1371/journal.pone.0222004 (PMC6760757; doi:10.1371/journal.pone.0222004)
Supplement: S2 Table — (DOCX) [file pone.0222004.s002.docx]

**S2 Table:** Fin whale *de novo* assembly quality information recovered using QUAST results on the pre and post scaffolded assembly.

| **Assembly feature** | **Post-scaffolding** | **Pre-scaffolding** |
| --- | --- | --- |
| # contigs (>=0bp) | 62,306 | - |
| # contigs (>=1000bp) | 59,639 | 494,216 |
| # contigs (>=5000bp) | 8,140 | 109,783 |
| # contigs (>=10000bp) | 5,706 | 24,430 |
| # contigs (>=25000bp) | 4,887 | 532 |
| # contigs (>=50000bp) | 4,315 | 1 |
| Total length (>=0bp) | 2,462,783,758 | - |
| Total length (>=1,000bp) | 2,460,448,386 | 1,839,189,252 |
| Total length (>=5,000bp) | 2,364,967,838 | 918,744,102 |
| Total length (>=10,000bp) | 2,348,864,187 | 334,788,093 |
| Total length (>=25,000bp) | 2,336,355,876 | 15,479,658 |
| Total length (>=50,000bp) | 2,315,286,921 | 57,005 |
| # contigs | 59,639 | 494,216 |
| Largest contig | 6,825,338 | 57,005 |
| Total length | 2,460,448,386 | 1,839,189,252 |
| N50 | 871,369 | 4,995 |
| N75 | 416,530 | 2,823 |
| L50 | 802 | 109,954 |
| L75 | 1,821 | 232,559 |
| #N's per 100 kbp | 17,749 | 9 |
