# Supplementary material for: Genomic analyses reveal an absence of contemporary introgressive admixture between fin whales and blue whales, despite known hybrids
Source: PLoS One. 2019 Sep 25;14(9):e0222004. doi: 10.1371/journal.pone.0222004 (PMC6760757; doi:10.1371/journal.pone.0222004)
Supplement: S3 Table — (DOCX) [file pone.0222004.s003.docx]

**S3 Table:** BUSCO scores of the fin whale genome assembly when using the BUSCOv3 mammal dataset.

| **Category** | **Number of BUSCO** | **Percentage** |
| --- | --- | --- |
| Complete BUSCOs (C) | 3,630 | 88.4 |
| Complete and single-copy BUSCOs (S) | 3,596 | 87.6 |
| Complete and duplicated BUSCOs (D) | 34 | 0.8 |
| Fragmented BUSCOs (F) | 285 | 6.9 |
| Missing BUSCOs (M) | 189 | 4.7 |
| Total BUSCO groups searched | 4,104 |  |
