# Supplementary material for: Genomic analyses reveal an absence of contemporary introgressive admixture between fin whales and blue whales, despite known hybrids
Source: PLoS One. 2019 Sep 25;14(9):e0222004. doi: 10.1371/journal.pone.0222004 (PMC6760757; doi:10.1371/journal.pone.0222004)
Supplement: S4 Table — (DOCX) [file pone.0222004.s004.docx]

**S4 Table**: Fin whale genome repeat profile.

| **Repeat type** | **De novo repeats (%)** | **Model based repeats (%)** | **Total (%)** |
| --- | --- | --- | --- |
| Total | 29.19 | 2.3 | 31.49 |
| SINEs | 4.55 | 0.04 | 4.59 |
| LINEs | 16.68 | 1.13 | 17.81 |
| LTR elements | 4.78 | 0.62 | 5.4 |
| DNA elements | 3.08 | 0.31 | 3.39 |
| Unclassified | 0.05 | 0.17 | 0.22 |
| Small RNA | 1.44 | 0.04 | 1.48 |
| Satellites | 0.02 | 0.02 | 0.04 |

**S5 Table:** Mapping statistics of all individuals included in the present study mapped to both our fin whale assembly and the previously published bowhead whale genome. Coverage was calculated using the total number of bp in each assembly excluding missing data (Fin whale - 2,025,416,608bp, Bowhead whale - 2,099,136,199bp).

| **Species** | **Raw read pairs** | **Trimmed reads** | **Unique reads mapping to fin whale** | **Total bp mapped** | **Coverage** | **Unique reads mapping to bowhead** | **Total bp mapped** | **Coverage** |
| --- | --- | --- | --- | --- | --- | --- | --- | --- |
| Fin | 578,742,088 | 578,619,831 | 453,595,057 | 44,352,066,368 | 21.90 | 455,598,362 | 44,641,333,295 | 21.27 |
| Humpback | 270,191,526 | 270,191,477 | 317,073,911 | 38,536,877,667 | 19.03 | 324,761,268 | 39,682,562,722 | 18.90 |
| Blue | 559,522,507 | 559,159,659 | 655,499,901 | 64,970,766,372 | 32.08 | 672,861,318 | 66,976,489,295 | 31.91 |
| Bowhead | 738,037,359 | 737,509,184 | 850,540,166 | 84,507,624,823 | 41.72 | 914,723,543 | 91,836,799,463 | 43.75 |

**Supporting figures**

**
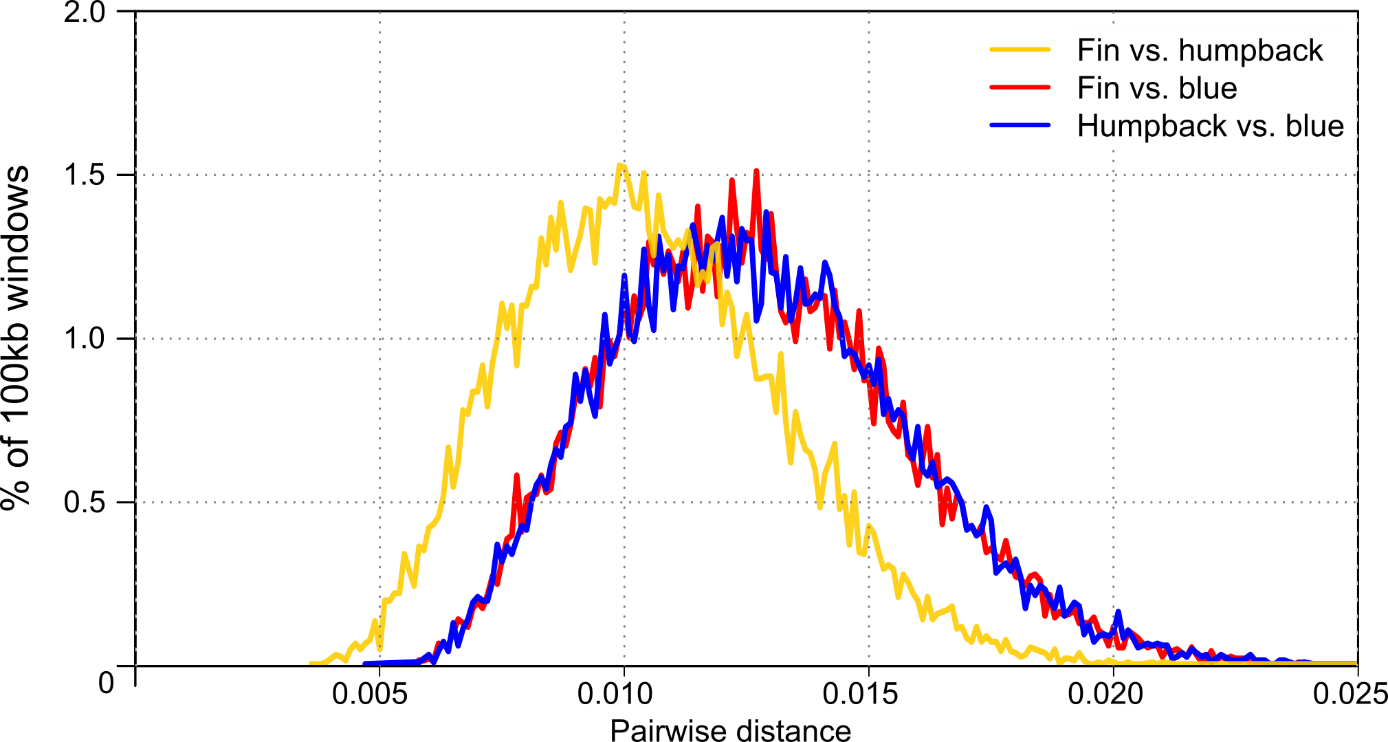
**

**S1 Figure:** Sliding window pairwise comparisons produced using the bowhead whale as the mapping reference. 100kb non-overlapping window identity-by-state, pairwise distance comparisons between each species pair.

**S2 Figure:** hPSMC plot based on the demographic history of pseudodiploid sequences constructed from different species pairs using the bowhead whale as the mapping reference.


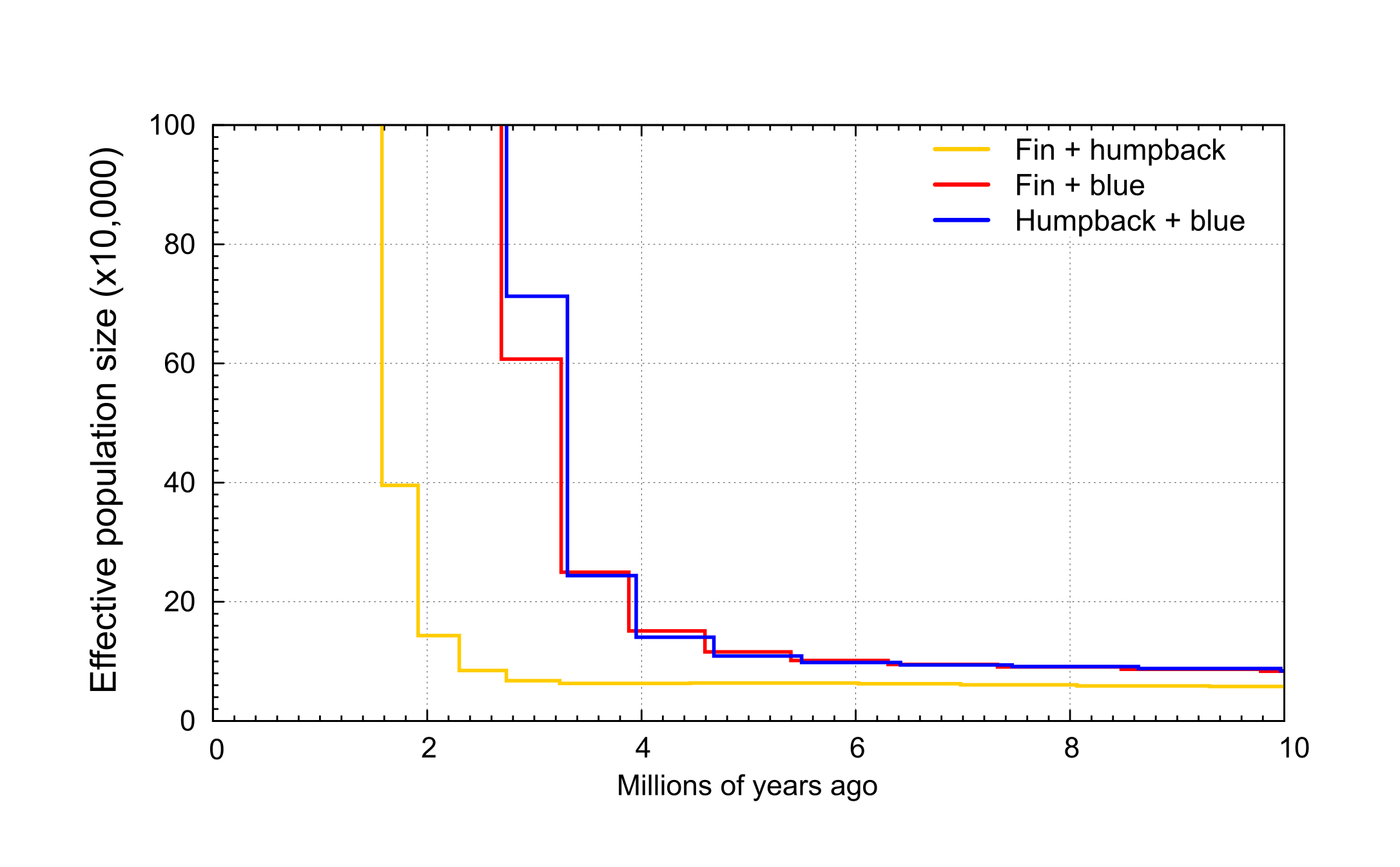


**
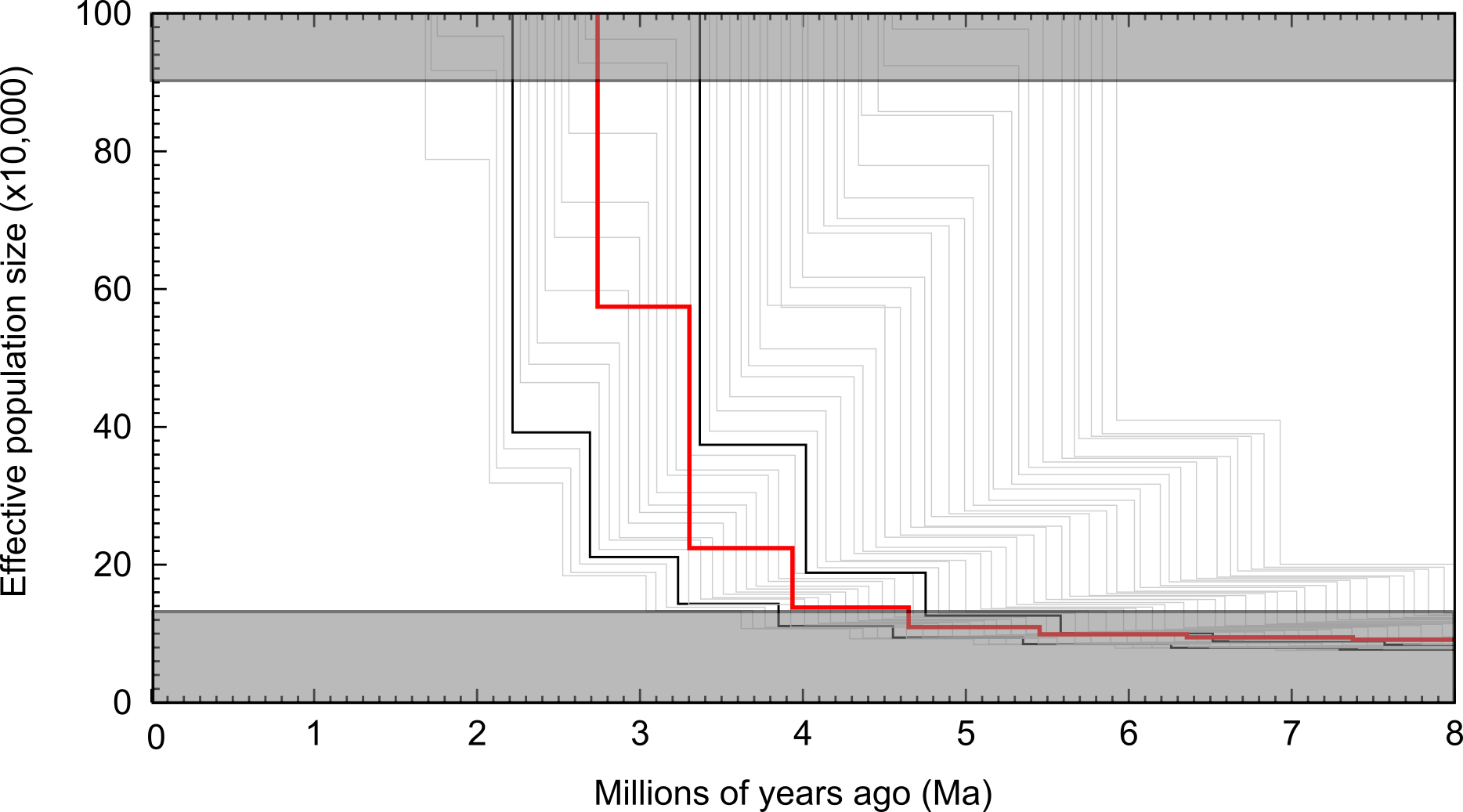
**

**S3 Figure:** hPSMC plot between the fin and blue whale and simulations of various different divergence times. Greyed out regions represent 1.5x and 10x the pre-divergence effective population size, grey lines represent the simulated data in 100kya intervals starting from 1Ma and ending at 5Ma, black line represents the simulations closest to the real data without overlapping it, red line represents the hPSMC result.


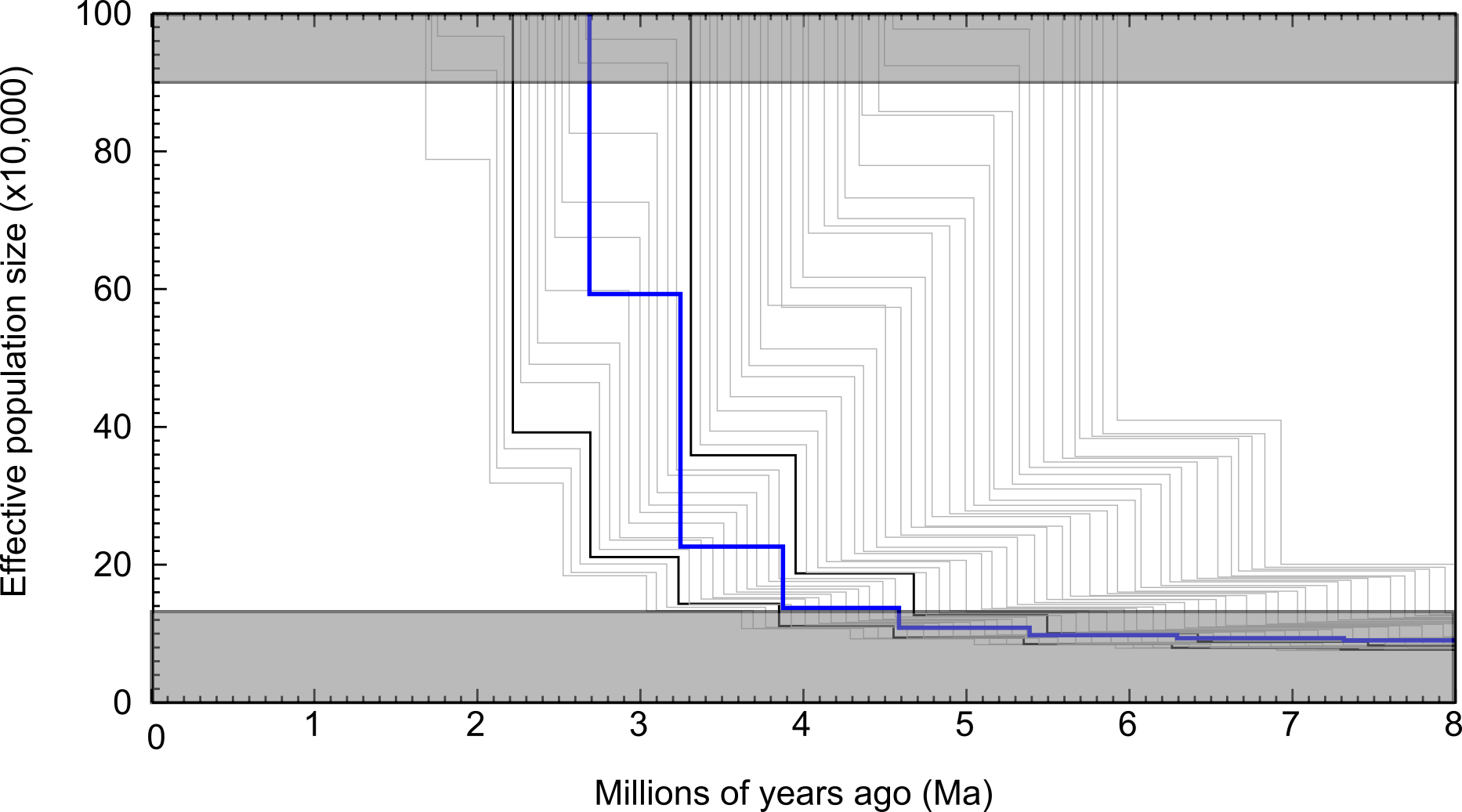


**S4 Figure:** hPSMC plot between the humpback and blue whale and simulations of various different divergence times. Greyed out regions represent 1.5x and 10x the pre-divergence effective population size, grey lines represent the simulated data in 100kya intervals starting from 1Ma and ending at 5Ma, black line represents the simulations closest to the real data without overlapping it, green line represents the hPSMC result.


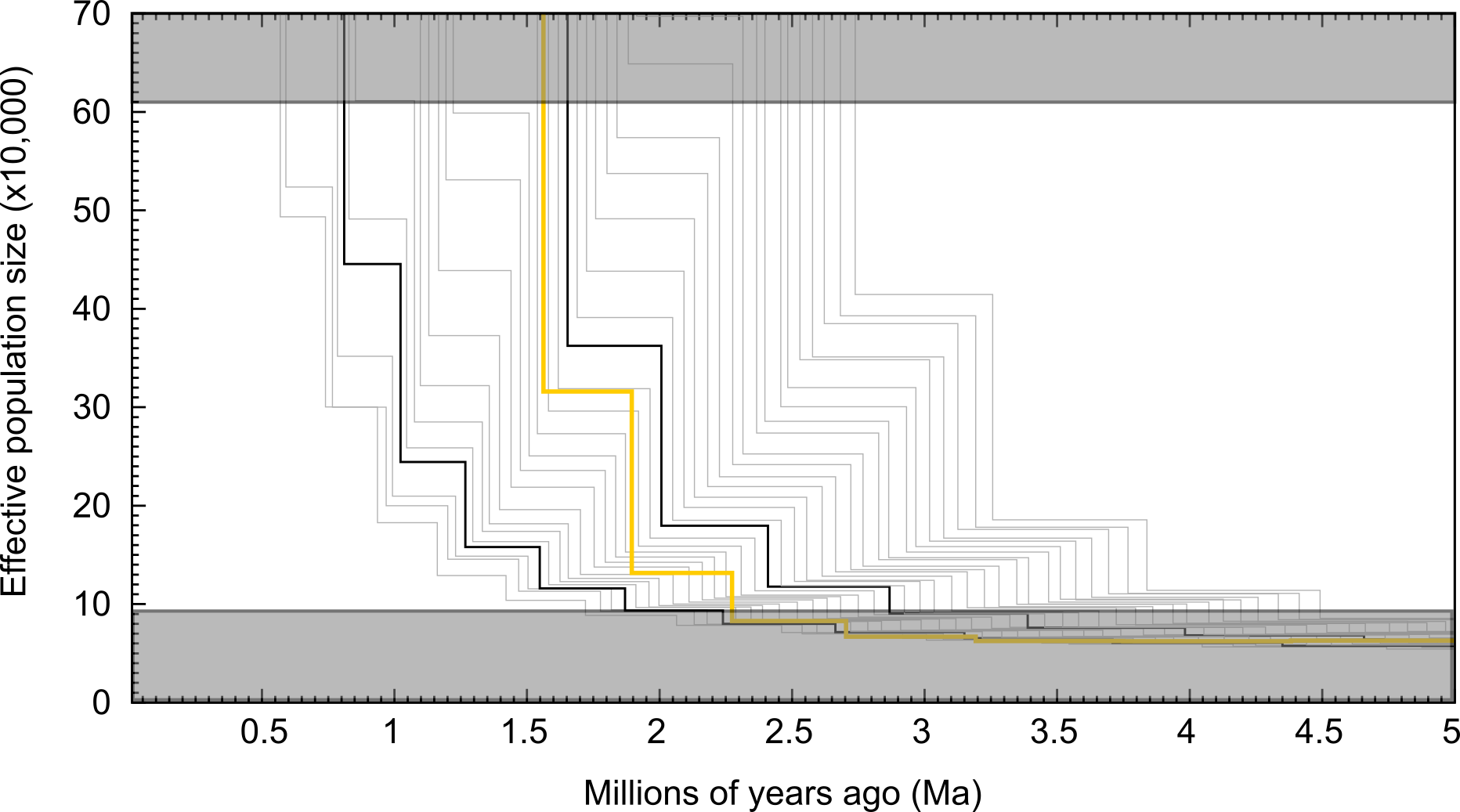


**S5 Figure:** hPSMC plot between the fin and humpback whale and simulations of various different divergence times. Greyed out regions represent 1.5x and 10x the pre-divergence effective population size, grey lines represent the simulated data in 100kya intervals starting from 0Ma and ending at 3Ma, black line represents the simulations closest to the real data without overlapping it, blue line represents the hPSMC result.
