# Supplementary material for: Genomic analyses reveal an absence of contemporary introgressive admixture between fin whales and blue whales, despite known hybrids
Source: PLoS One. 2019 Sep 25;14(9):e0222004. doi: 10.1371/journal.pone.0222004 (PMC6760757; doi:10.1371/journal.pone.0222004)
Supplement: S5 Table — Coverage was calculated using the total number of bp in each assembly excluding missing data (Fin whale—2,025,416,608bp, Bowhead whale—2,099,136,199bp). (DOCX) [file pone.0222004.s005.docx]

**S5 Table:** Mapping statistics of all individuals included in the present study mapped to both our fin whale assembly and the previously published bowhead whale genome. Coverage was calculated using the total number of bp in each assembly excluding missing data (Fin whale - 2,025,416,608bp, Bowhead whale - 2,099,136,199bp).

| **Species** | **Raw read pairs** | **Trimmed reads** | **Unique reads mapping to fin whale** | **Total bp mapped** | **Coverage** | **Unique reads mapping to bowhead** | **Total bp mapped** | **Coverage** |
| --- | --- | --- | --- | --- | --- | --- | --- | --- |
| Fin | 578,742,088 | 578,619,831 | 453,595,057 | 44,352,066,368 | 21.90 | 455,598,362 | 44,641,333,295 | 21.27 |
| Humpback | 270,191,526 | 270,191,477 | 317,073,911 | 38,536,877,667 | 19.03 | 324,761,268 | 39,682,562,722 | 18.90 |
| Blue | 559,522,507 | 559,159,659 | 655,499,901 | 64,970,766,372 | 32.08 | 672,861,318 | 66,976,489,295 | 31.91 |
| Bowhead | 738,037,359 | 737,509,184 | 850,540,166 | 84,507,624,823 | 41.72 | 914,723,543 | 91,836,799,463 | 43.75 |
