# Supplementary material for: Genomic analyses reveal an absence of contemporary introgressive admixture between fin whales and blue whales, despite known hybrids
Source: PLoS One. 2019 Sep 25;14(9):e0222004. doi: 10.1371/journal.pone.0222004 (PMC6760757; doi:10.1371/journal.pone.0222004)
Supplement: S1 Fig — 100kb non-overlapping window identity-by-state, pairwise distance comparisons between each species pair. (DOCX) [file pone.0222004.s006.docx]

**
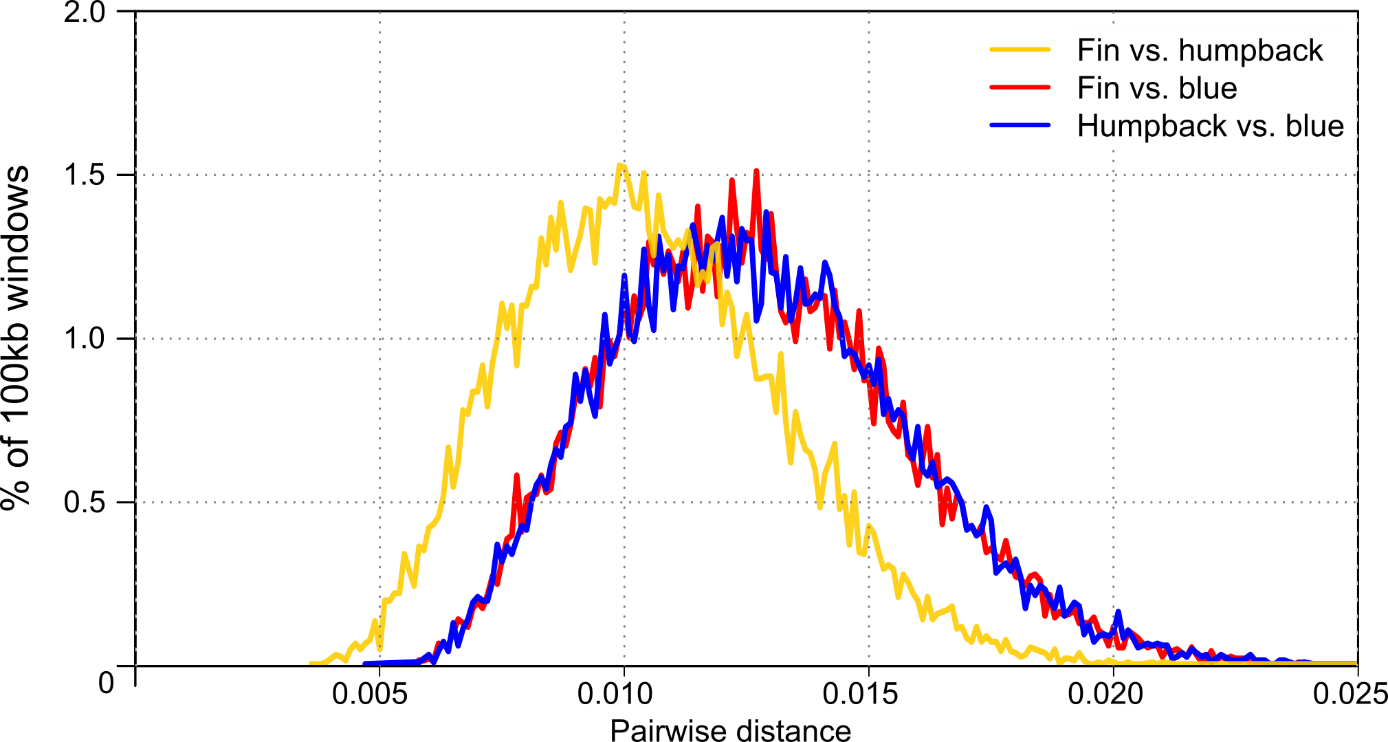
**

**S1 Figure:** Sliding window pairwise comparisons produced using the bowhead whale as the mapping reference. 100kb non-overlapping window identity-by-state, pairwise distance comparisons between each species pair.
