# Supplementary material for: Genomic analyses reveal an absence of contemporary introgressive admixture between fin whales and blue whales, despite known hybrids
Source: PLoS One. 2019 Sep 25;14(9):e0222004. doi: 10.1371/journal.pone.0222004 (PMC6760757; doi:10.1371/journal.pone.0222004)

**S2 Figure:** hPSMC plot based on the demographic history of pseudodiploid sequences constructed from different species pairs using the bowhead whale as the mapping reference.


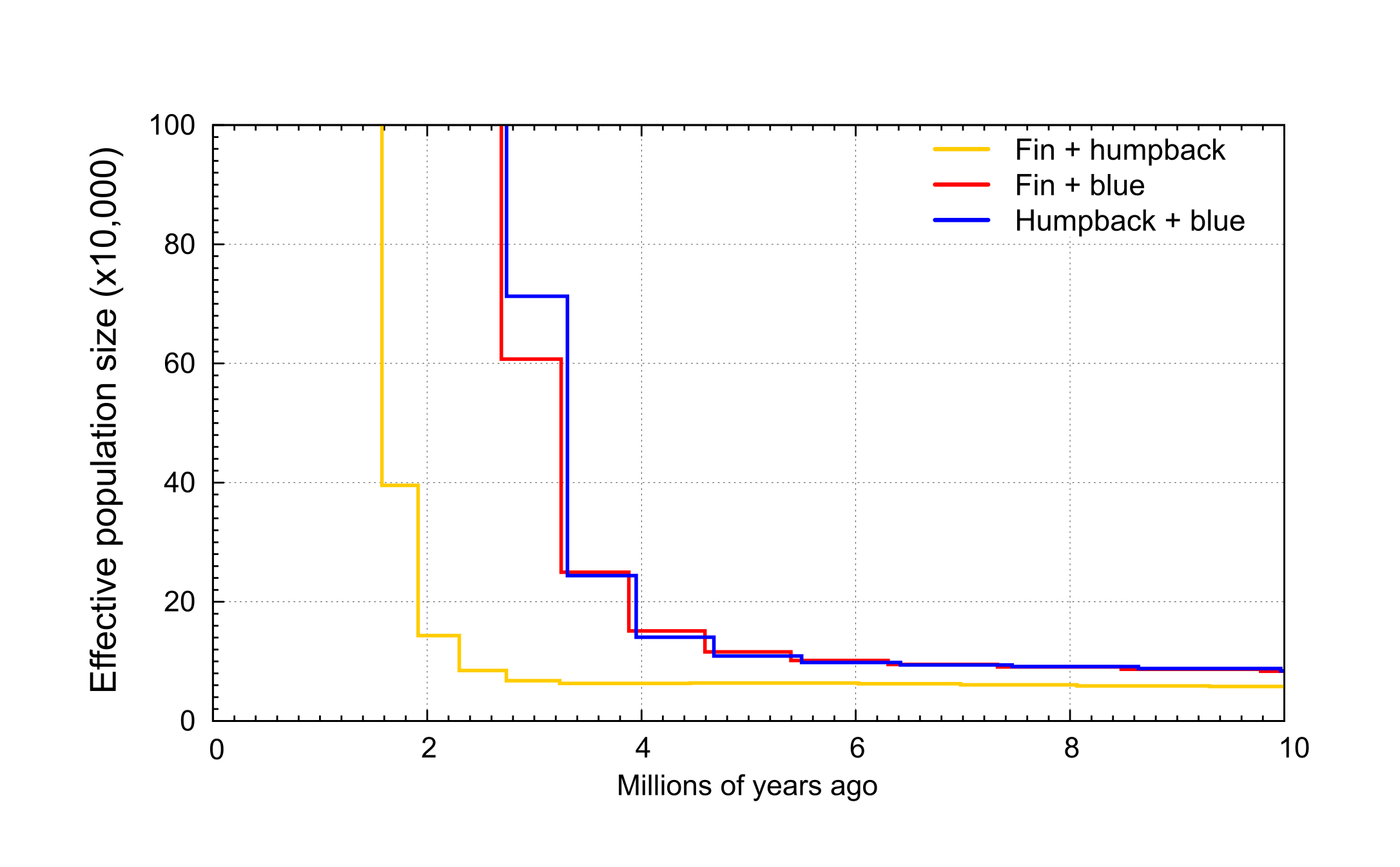

Supplement: S2 Fig — (DOCX) [file pone.0222004.s007.docx]
