# Supplementary material for: Genomic analyses reveal an absence of contemporary introgressive admixture between fin whales and blue whales, despite known hybrids
Source: PLoS One. 2019 Sep 25;14(9):e0222004. doi: 10.1371/journal.pone.0222004 (PMC6760757; doi:10.1371/journal.pone.0222004)
Supplement: S3 Fig — Greyed out regions represent 1.5x and 10x the pre-divergence effective population size, grey lines represent the simulated data in 100kya intervals starting from 1Ma and ending at 5Ma, black line represents the simulations closest to the real data without overlapping it, red line represents the hPSMC result. (DOCX) [file pone.0222004.s008.docx]

**
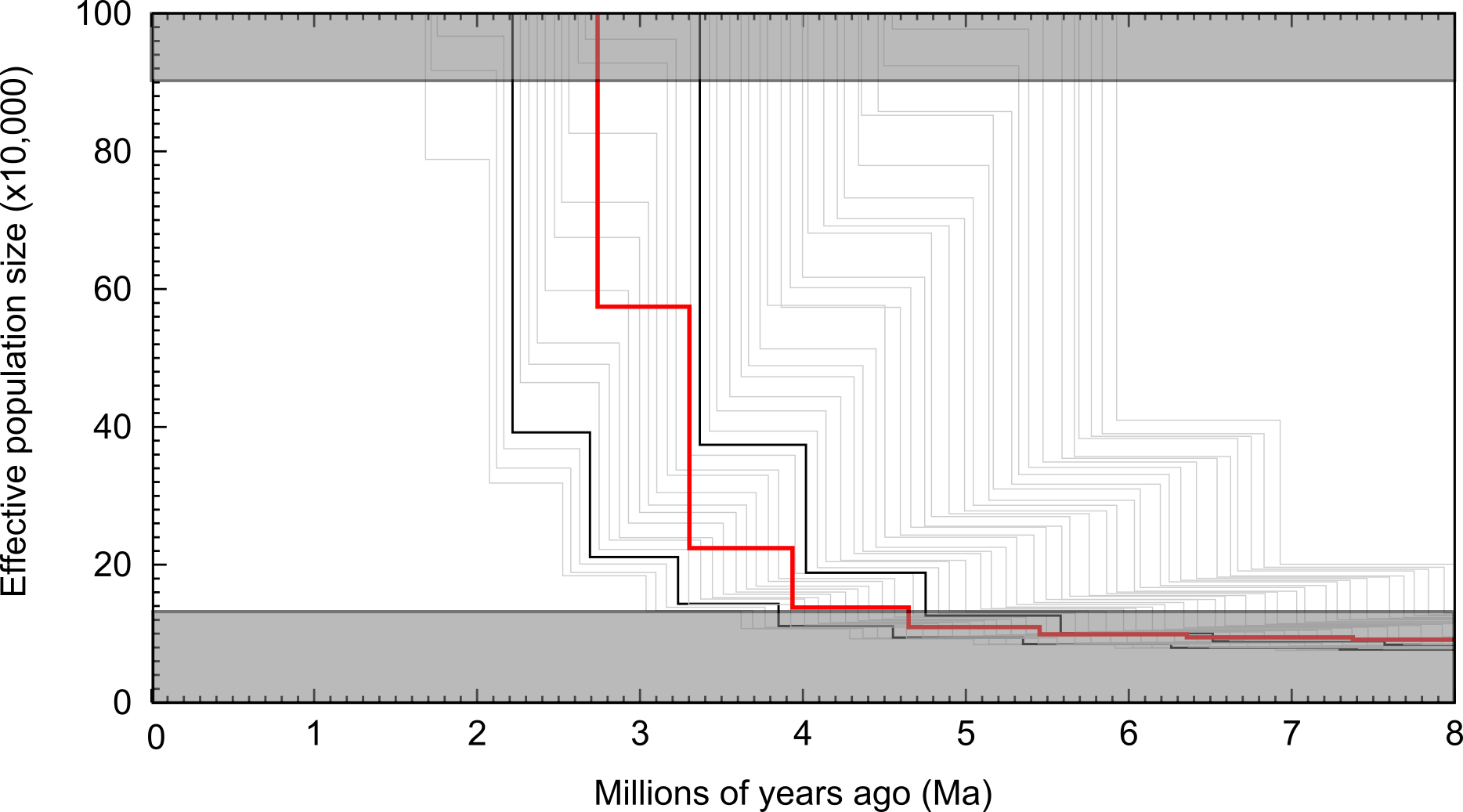
**

**S3 Figure:** hPSMC plot between the fin and blue whale and simulations of various different divergence times. Greyed out regions represent 1.5x and 10x the pre-divergence effective population size, grey lines represent the simulated data in 100kya intervals starting from 1Ma and ending at 5Ma, black line represents the simulations closest to the real data without overlapping it, red line represents the hPSMC result.
